# Characterization of the complete plastid genome sequence of *Breynia fruticosa* (L.) Müll.Arg. (Phyllanthaceae), a traditional Chinese medicine plant

**DOI:** 10.1080/23802359.2020.1828002

**Published:** 2020-10-07

**Authors:** Zengliang Zhou, Ruilan Cao, Dongnan Hu, Juan Liu

**Affiliations:** Jiangxi Provincial Key Laboratory of Silviculture, College of Forestry, Jiangxi Agricultural University, Nanchang, China

**Keywords:** Breynia, medicine plant, chloroplast genome, phylogenetics

## Abstract

*Breynia fruticosa* (L.) Müll.Arg. is a well-known folk medicinal plant and found abundantly in South China. The complete chloroplast genome of *B. fruticosa* reported firstly here was 155,630 bp in length, including a large single-copy region with 85,065 bp (LSC), a small single-copy region with 19,441 bp (SSC) and a pair of inverted repeats with 25, 562 bp (IRa and IRb). The plastome was comprised of 112 distinct genes, with 78 protein coding genes, four ribosomal RNA genes and 30 transfer RNA genes. The overall GC content of *B. fruticose* chloroplast genome was 36.7%. Phylogenetic analysis revealed that *B. fruticosa* was closely related to *Glochidion fruticosa*.

The genus *Breynia* (Phyllanthaceae) contains about 26 species, and the majority of which are distributed in Southeast Asia (Qiu et al. 1996). Four species of *Breynia* (*B. officinalis, B. fruticosa, B. vitis-idaea* and *B. rostrata*) in China are widely used as Chinese traditional medicine (Xie and Xie 1996; Sasaki et al. 2018). For example, *B. fruticosa* (L.) Müll.Arg. f., which is widely distributed in South China, is a folk medicine for treatments of gastroenteritis, sore throat, chronic bronchitis and wounds (Meng et al. 2010; Liu et al. 2011; He et al. 2019). Many previous researches have focused on chemical ingredients and medicine activities of *Breynia* plants (Song 1999; Liu et al. 2011; Sasaki et al. 2018), however there are limited studies on the genetic resource of *Breynia* species. In this study, the complete plastid genome of *B. fruticosa* was sequenced and assembled for the first time, and the data obtained here would provide genomic resources of *Breynia* species for further research.

We collected fresh leaves of of *B. fruticosa* in Guanghzou, Guangdong province (China; N 23°12′26.02″, E 113°21′5.13″ and deposited the voucher specimen (*YAO-YGGD2019040601*) in the Herbarium of South China Botanical Garden, Chinese Academy of Sciences (IBSC). Total genomic DNA of *B. fruticosa* was extracted from its fresh leaves materials using modified CTAB method (Doyle and Doyle 1987). Genomic sequencing was performed on the Illumina HisSeq 2500 Sequencing System. Software GetOrganelle (Jin et al. 2018) was employed to assemble the reads and PGA (Qu et al. 2019) was used to annotate all the plastid genes. The annotated plastid genomic sequence of *B. fruticosa* is available in the GenBank with the accession number MT863745.

The plastid genome of *B. fruticosa* was 155,630 bp in length, including a large single-copy region with 85,065 bp (LSC), a small single-copyregion with 19,441 bp (SSC), and a pair of inverted repeats with 25,562 bp (IRa and IRb). The overall GC content of *B. fruticose* chloroplast genome was 36.7% (LSC: 34.5%, SSC: 30.3%, IRs: 43.0%). The annotated genome predicted 112 distinct genes, including 78 protein coding genes, four ribosomal RNA genes (rrn16, rrn23, rrn4.5, rrn5) and 30 transfer RNA genes. Within the annotated genes, there were 17 genes in the IR regions, with 6 protein-coding genes (*rpl2*, *rpl23*, *ycf2*, *ndhB*, *rps7*, *rps12*), four ribosomal RNA genes (*rrn16*, *rrn23*, *rrn4.5*, *rrn5*) and seven transfer RNA genes (*trnI-CAU*, *trnL-CAA*, *trnV-GAC*, *trnI-GAU*, *trnA-UGC*, *trnR-ACG*, *trnN-GUU*).

The maximum likelihood (ML) phylogenetic tree was constructed using RAxML –HPC on the CIPRES cluster. A combined sequence matrix was emoloyed, including all the 78 coding genes of *B. fruticosa* and Phyllanthaceae and Euphorbiaceae from 9 plastid genomes downloaded from the website of NCBI (www.ncbi.nlm.nih.gov). Sequences were aligned using MAFFT v7.017 plugin in Geneious R11.1.5. The GTR + G model and the default number of rate categories were set. The support values of phylogenetic nodes were conducted on the basis of a rapid bootstrap (BS) analysis with 1000 replicates. The phylogenetic result (Figure 1) showed that *Breynia* was sister to the genus *Glochidion* with strong support value (MLBS = 100%). The complete plastid genome of *B. fruticosa* reported here would provide a useful genetic resource on genetic diversity and conservation of *Breynia* species.

**Figure 1.**
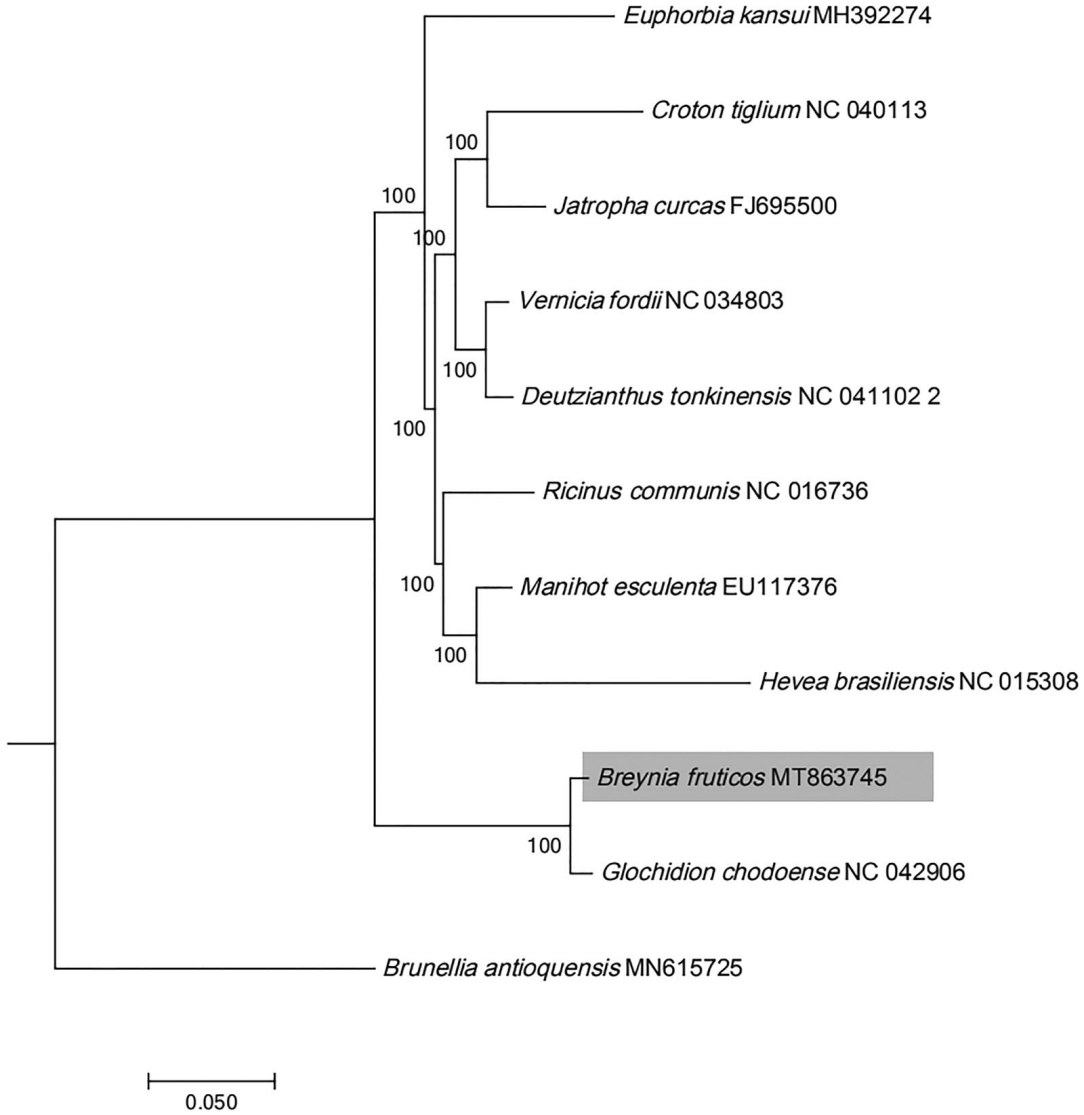
Maximum likelihood tree of Phyllanthaceae and Euphorbiaceae was inferred from 9 plastomes. Bootstrap values were indicated beyond each node.

## Data Availability

The data that support the findings of this study are openly available in GenBank of NCBI at https://www.ncbi.nlm.nih.gov, reference number MT863745.
